# Secondhand smoke exposure and associated factors among city residents living in multiunit housing in Bangladesh

**DOI:** 10.1371/journal.pone.0291746

**Published:** 2023-09-21

**Authors:** Md. Golam Kibria, Ahmed Hossain, Taslima Islam, Kazi Rakibul Islam, H. M. Miraz Mahmud, Mohammad Hayatun Nabi, Mohammad Delwer Hossain Hawlader

**Affiliations:** 1 Department of Research, Centre for Development Action, Dhaka, Bangladesh; 2 Health Services Administrations, College of Health Sciences, University of Sharjah, Sharjah, UAE; 3 Department of Monitoring, Evaluation and Learning, Social Development Foundation, Dhaka, Bangladesh; 4 Department of Public Health, Northern University Bangladesh, Dhaka, Bangladesh; 5 Department of Research & Evaluation, Bangladesh Center for Communication Programs, Dhaka, Bangladesh; 6 Department of Public Health, North South University, Dhaka, Bangladesh; Severance Hospital, Yonsei University College of Medicine, REPUBLIC OF KOREA

## Abstract

**Background:**

Secondhand smoke (SHS) poses a high health risk to those living in multiunit housing (MUH) since it can easily spread from unit to unit and throughout the building’s communal areas. MUH residents in Bangladesh are particularly vulnerable to SHS due to the absence of smoking restrictions within a housing complex. Therefore, this study aimed to assess the prevalence of SHS exposure and its associated factors among MUH residents living in seven divisional cities of Bangladesh- Dhaka, Chattogram, Rajshahi, Khulna, Sylhet, Barishal, and Rangpur.

**Methods:**

From April 2019 to November 2019, a cross-sectional survey was conducted with 616 MUH residents aged 18 or older who had been residing in MUH for at least two years in the seven divisional cities of Bangladesh. A multivariable logistic regression model was performed to determine the associated factors of SHS exposure.

**Results:**

In MUH complexes, more than half (54.9%) of the 616 respondents were exposed to SHS. The key factors positively associated with SHS exposure were females (aOR: 1.8, 95% CI:1.236–2.681), residents with a low monthly family income (aOR: 1.9, 95% CI: 1.162–3.220), those whose family members smoked (aOR: 2.4, 95% CI: 1.537–3.746), and Dhaka city residents (aOR: 1.9, 95% CI: 1.013–3.440).

**Conclusions:**

This study revealed a high prevalence of SHS exposure among Bangladeshi MUH residents. Therefore, a smoking ban is needed in and around MUH complexes to protect non-smoking residents from SHS exposure.

## Introduction

It is widely recognized that exposure to secondhand smoke (SHS) poses serious health risks for individuals around the world. Both the mainstream smoke breathed by smokers and the sidestream smoke emitted into the environment by burning cigarettes and other tobacco products combine to create SHS [1]. Tobacco smoke contains more than 7,000 chemical components, of which at least 250 are known to be harmful to human health [2]. There is no safe level of SHS exposure, and even brief exposure can affect human health seriously [3]. A large body of epidemiological research has established a link between SHS exposure and increased morbidity and mortality. SHS exposure is a major risk factor for heart disease, stroke, and lung cancer in adults [3–5]. SHS exposure results in adverse health outcomes in women of reproductive age [6]. Children exposed to SHS are at higher risk of bronchitis, pneumonia, asthma, ear infections, and sudden infant death syndrome (SIDS) than those not exposed to SHS [7, 8]. There are an estimated 1.3 billion tobacco users worldwide, and over 80% of them live in low- and middle-income countries (LMCs) [9]. Bangladesh, a lower middle-income country, is one of the world’s largest tobacco consumption countries with a smoking prevalence of 18.0% among the adult population [10]. Globally, tobacco use causes more than 8 million premature deaths every year, of which around 1.2 million are attributable to SHS exposure [9]. A recent study found that in Bangladesh, about 25,000 people die annually due to SHS exposure, and the cost of productivity losses from these premature deaths is estimated at BDT 39.27 billion [11].

Bangladesh is one of the most densely populated countries in the world with a 38.9% urban population [12]. To meet the housing needs of an increasing urban population, multiunit housing (MUH) has become the country’s largest housing option. MUH is a residential building consisting of at least two separate units with shared areas, including common building entrance, elevators, stairs, basements, lobbies, parking areas, waiting spaces, and roofs. MUH residents in the country are particularly vulnerable to SHS exposure because there is no provision to restrict smoking at home and inside residential buildings. However, in recent years, some developed nations, such as the United States of America, Canada, and Australia have adopted smoke-free housing policies to protect their MUH residents from involuntary exposure to SHS [13–15]. Existing evidence indicates that the implementation of smoke-free housing policies not only reduces SHS exposure but also increases smoking cessation among MUH residents [16].

Bangladesh is the signatory of the WHO Framework Convention on Tobacco Control (FCTC) and enacted the ‘Smoking and Tobacco Products Usage (Control) Act 2005’ in line with the FCTC [17]. The definition of public place and public transport was widened through an amendment of the Act in 2013, but there was no provision in the amendment to restrict smoking in the common spaces of a multiunit housing complex. Furthermore, there is a national housing policy that has not restricted tobacco smoking at home and inside housing complexes [18]. Consequently, housing residents can smoke anywhere in MUH complexes.

In Bangladesh, 39.0% of adults (equivalent to 40.8 million) are exposed to SHS at home due to smoking by family members and visitors [10]. This prevalence does not represent the overall prevalence of SHS exposure in a residential setting because this refers to only smoking at home. Available literature shows that housing residents are also exposed to SHS from other sources like neighbouring units, common areas, and adjacent buildings [19, 20]. Therefore, this study aimed to assess the prevalence of SHS exposure among city residents at home and from neighbouring units, common areas, and next buildings in the seven divisional cities of Bangladesh–Dhaka, Chattogram, Rajshahi, Khulna, Sylhet, Barishal, and Rangpur as well as identify its associated factors.

## Materials and methods

### Study design and participants

During the months of April and November of 2019, MUH residents from the seven divisional cities participated in a cross-sectional study. Adults (18+) with a minimum 2-year residency in multiunit housing were eligible to participate. Pregnant women and people with speech and/or hearing difficulties were not included in this study.

### Measures

#### Basic information

Self-reported basic variables included sex (male or female), age (18–39, 40–59, or ≥60 years), marital status (single or ever married), education level (primary, secondary, higher secondary, or tertiary), occupation (job holder, business, student, retired, or housewife), religion (Muslim, Hindu, or Buddhist), monthly family income (<50,000 BDT, 50,000–99,000 BDT, or ≥100,000 BDT), smoking status (smoker or non-smoker), smoking by family members (yes or no), length of stay at home (≤12 hours or >12 hours), type of housing complex (private or government), place of residence (Dhaka city, Chattogram city, Rajshahi city, Khulna city, Sylhet city, Barishal city, or Rangpur city). The smoking status of the respondents was determined by asking, “On how many days during the past 30 days, did you smoke cigarettes/bidis?” Those who smoked cigarettes/bidis on at least one day during the past 30 days were categorized as ‘smokers’, and on the other hand, those who did not smoke cigarettes/bidis during the past 30 days were categorized as ‘non-smokers’ [21].

#### SHS exposure

Self-reported SHS exposure was assessed using the question, “On how many days during the past 30 days, did you get the smell of cigarettes/bidis from the following places: (a) your own flats, (b) next flats, (c) common spaces and (d) next buildings?” Those who reported any number between 1 and 30 days were considered exposed to SHS from the particular place [22]. In this study, overall SHS exposure within MUH complexes was the outcome variable and was defined as exposure from at least one of these places—own flats, next flats, common spaces and next buildings during the past 30 days.

#### Sample size calculation and distribution

The minimum required sample size for this study was calculated using a single population proportion formula, n = $\frac{\left(z_{\alpha /2})2p(1-p\right)}{e^{2}}$ [23], where n = desired sample size, z = standard normal deviate = 1.96 at 95% confidence interval, p = prevalence of SHS exposure (unknown) among city residents living in MUH in the divisional cities of Bangladesh = 50%, and e = margin of error = 4%. This yielded a sample size of 600.

The number of MUH complexes in Dhaka city is much higher than that in any other city in the country. Therefore, we allocated about one-third of the sample to Dhaka city and distribute the remaining two-thirds among the six cities equally. There was no published list of MUH complexes in Bangladesh. Taking budget constraints into account, we randomly selected ten wards from each of the seven cities and prepared a city-wise list of all MUH complexes in the selected wards. We did not include solely-owned MUH complexes in the list because those did not have a housing management committee. The list included 1,067 MUH complexes from Dhaka city, 210 from Chattogram city, 120 from Rajshahi city, 95 from Khulna city, 90 from Sylhet city, 80 from Barishal city, and 105 from Rangpur city. With a plan to enroll three to five respondents from each MUH complex, we randomly selected 60 complexes from Dhaka city and 20 complexes from each of the six cities. Finally, we interviewed a total of 616 respondents, including 214 from Dhaka city and 67 from each of the six cities.

#### Training and pretesting

For data collection for this study, we recruited six teams of experienced field staff with a social science background. Each team consisted of three members—one male data collector, one female data collector, and one male field supervisor. Before data collection, they were given two-day training so that they could thoroughly understand the objectives and methodology of the study and the survey questionnaire. They were also taught the techniques of rapport building, environment control, maintaining neutrality, and obtaining informed consent from the respondents. However, a semi-structured questionnaire was first developed in English, and it was forward-translated into the local language Bengali and back-translated into English by two bilingual translators to ensure the accuracy of the contents [24]. Following the training, the Bengali version questionnaire was pretested among 25 respondents in similar study settings to determine its clarity, practicality, and relevance to the study participants. The questionnaire was finalized after necessary modifications based on the findings of the pretest.

#### Fieldwork

First of all, the field supervisors approached the housing management committees of the selected complexes with an official letter seeking permission for data collection. After obtaining permission, the data collectors accompanied by the field supervisors visited the housing complexes and informed the guards on duty about the purpose of their visit. The guards introduced the data collectors with the residents available in their units via intercom, and subsequently the data collectors explained the objectives of the study to them. Those who consented to participate in the study were met by the data collectors and interviewed using a semi-structured survey questionnaire. A total of three to five residents from each MUH complex, including one resident from each unit were purposively selected for the face-to-face interviews. In case of two-unit MUH complexes, we recruited a maximum of two residents from each unit. These face-to-face interviews were held either in the respondents’ houses or in the waiting areas of the complexes during the daytime on both weekdays and weekends. An interview lasted for approximately 20 minutes. Each completed questionnaire was spot-checked by the field supervisors to assess the accuracy, completeness, and consistency of the data collected. Furthermore, the principal investigator and the co-investigator visited the study sites during data collection to ensure the data were collected properly. The respondents were not given any financial benefits for participating in the study.

#### Statistical analysis

Data analysis was performed using SPSS statistical software version 25.0. For descriptive statistics, frequencies and percentages were used to describe the basic characteristics of the respondents. Bivariate analyses were done using the chi-square test of independence for categorical variables. All independent variables were included in the binary logistic regression model, and the backward stepwise selection method was used to identify the factors associated with SHS exposure within MUH complexes. Associations between the outcome variable and the factors were presented as adjusted odds ratios (aOR) at 95% confidence intervals (CIs). Model fitness was assessed by using the Hosmer-Lemeshow test with a p-value of 0.509. Multicollinearity was checked by examining the standard errors (SEs) of regression coefficients in the logistic regression analyses. An SE with a range of 0.001 to 5.0 suggests that there is no multicollinearity among independent variables [25]. The SEs for the independent variables in our adjusted model were between 0.198 and 0.372, indicating the absence of multicollinearity. All statistics were tested using a two-sided test, and a p value of <0.05 was considered statistically significant.

#### Ethical considerations

Ethical clearance was obtained from the National Research Ethics Committee following the ethical guidelines of the Bangladesh Medical Research Council (BMRC) (Reference number: 25003092019). Written informed consent was taken from the respondents. The informed consent form clearly explained the aims and procedures of the study, the risks and benefits associated with participation, their right to voluntary participation and their right to withdraw from the study, and the anonymity and confidentiality of their data.

## Results

### Characteristics of the respondents

Table 1 displays the demographic breakdown of the 616 respondents. Males made up the majority (66.7%) of the respondents, and those between the ages of 40 and 59 made up the largest age group, followed by those between the ages of 18 and 39 (43.7%). By marital status, 85.4% were ever married, and 14.6% were single. About two-thirds of the respondents completed Bachelor’s degree or higher education, while about a third had secondary education. Most of the respondents (44.6%) were job holders, while 20.3% were housewives. In terms of monthly income, 44.0% of the respondents said their family made between 50,000 and 99,000 BDT per month, while 35.1% said they made less than 50,000 BDT per month. The majority (53.2%) of the respondents spent 12 hours or less a day at home during the past 30 days, 46.8% spent more than 12 hours a day at home during the past 30 days. By type of housing complex, more than three-fourths of the respondents resided in private MUH complexes. Over a third of the respondents (34.7%) were from Dhaka city, while 10.9% were from each of the other six divisional cities–Chattogram, Rajshahi, Khulna, Sylhet, Barishal, and Rangpur.

**Table 1:** Distribution of the respondents by characteristics (n = 616).

Variable	Category	Frequency	Percent
**Sex**	Male	411	66.7%
	Female	205	33.3%
**Age**	18–39 years	269	43.7%
	40–59 years	274	44.5%
	≥60 years	73	11.9%
**Marital status**	Single	90	14.6%
	Ever married	526	85.4%
**Education**	Primary	8	1.3%
	Secondary	34	5.5%
	Higher secondary	192	31.2%
	Bachelor’s degree or higher	382	62.0%
**Occupation**	Job holder	275	44.6%
	Business	98	15.9%
	Student	59	9.6%
	Retired	59	9.6%
	Housewife	125	20.3%
**Religion**	Muslim	563	91.4%
	Hindu	51	8.3%
	Buddhist	2	0.3%
**Monthly family income**	<50,000 BDT	216	35.1%
	50,000–99,000 BDT	271	44.0%
	≥100,00 BDT	129	20.9%
**Smoking status**	Smoker	121	19.6%
	Non-smoker	495	80.4%
**Smoking by family members**	Yes	136	22.1%
	No	480	77.9%
**Length of stay at home**	≤12 hours	328	53.2%
	>12 hours	288	46.8%
**Type of housing complex**	Private	471	76.5%
	Government	145	23.5%
**Place of residence**	Dhaka city	214	34.7%
	Chattogram city	67	10.9%
	Rajshahi city	67	10.9%
	Khulna city	67	10.9%
	Sylhet city	67	10.9%
	Barishal city	67	10.9%
	Rangpur city	67	10.9%

### SHS exposure from different places

**Fig 1:**
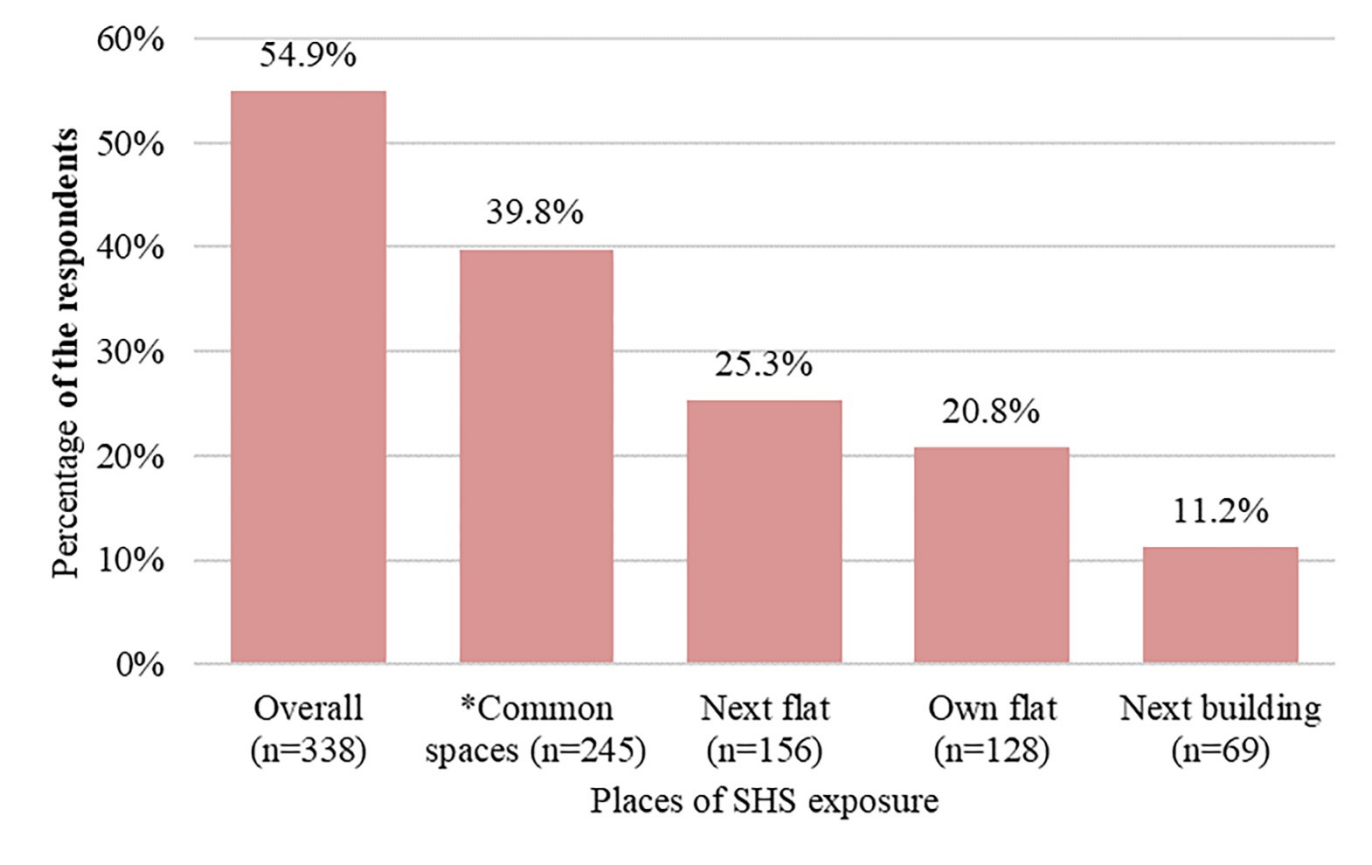
Prevalence of SHS exposure among MUH residents by place (n = 616). *Note*: *Common spaces in a multiunit housing complex include waiting spaces, parking areas, basements, stairs, roofs, doorways, and main entrances.

Fig 1 shows the level of SHS exposure from different places among MUH residents during the past 30 days. Overall, more than half of the respondents (54.9%) were exposed to SHS inside MUH complexes during the past 30 days. More specifically, about two-fifths of the respondents (39.8%) were exposed to SHS from common spaces within MUH complexes, followed by next flats (25.3%), own flats (20.8%), and next buildings (11.2%).

### Frequency of daily SHS exposure

**Fig 2:**
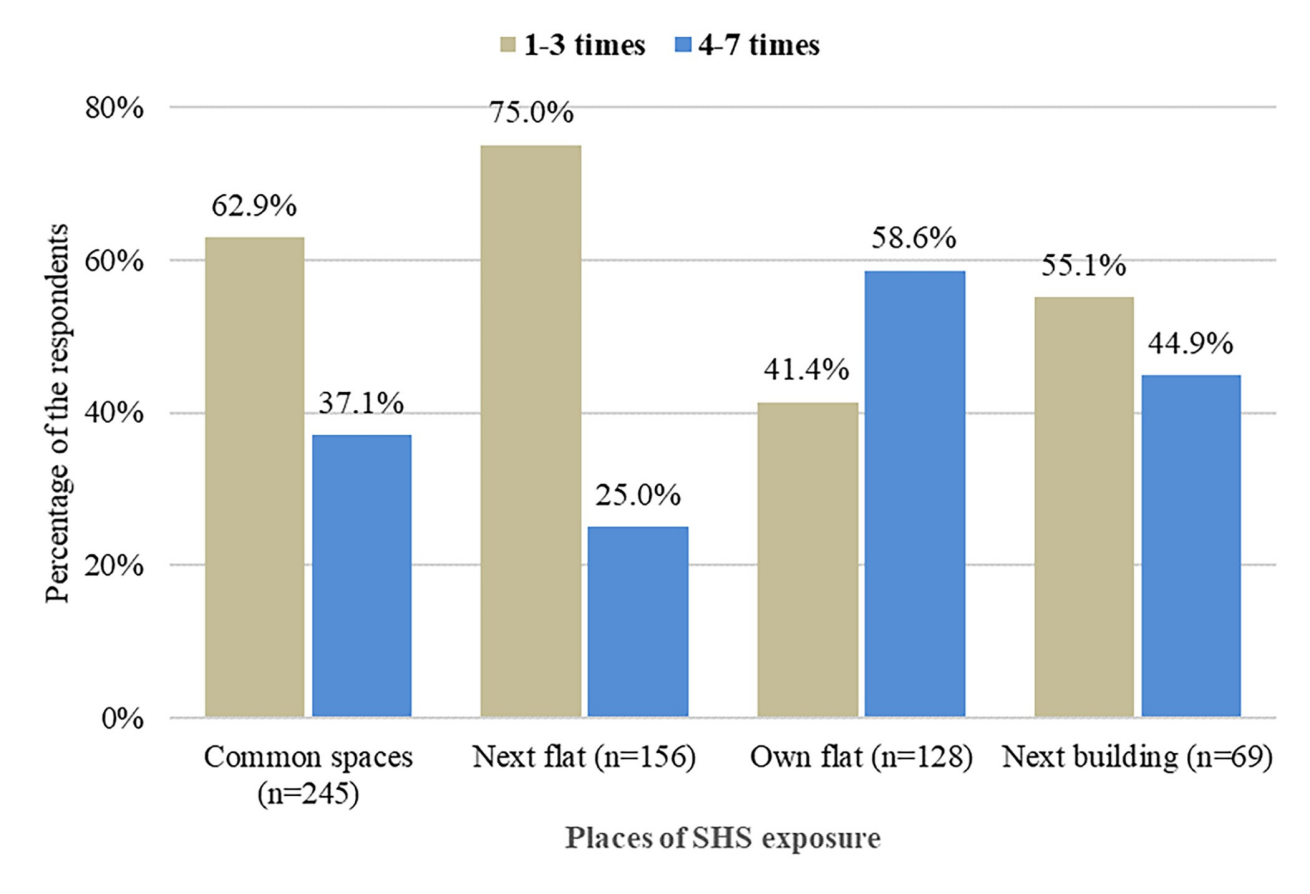
Frequency of daily SHS exposure among MUH residents (n = 616).

Fig 2 shows the frequency of daily SHS exposure from different places among MUH residents during the last 30 days. Among the respondents who reported daily SHS exposure in MUH complexes, most were exposed to SHS 1 to 3 times a day from next flats (75.0%), followed by common spaces (62.9%), and next buildings (55.0%). On the other hand, about three-fifths (58.6%) of the respondents who experienced daily SHS exposure in their own flats were exposed to SHS 4 to 7 times a day.

### Associations between SHS exposure and characteristics

According to the results of bivariate analysis shown in Table 2, sex, occupation, length of stay at home, smoking status, smoking by family members, and place of residence were found to be associated with SHS exposure in MUH complexes. SHS exposure was higher among the respondents who were female, housewives, stayed at home for more than 12 hours per day, were smokers, had smoking family members, and resided in Dhaka city.

**Table 2:** Association between SHS exposure and the respondents’ characteristics (n = 616).

Variable	SHS exposure in the MUH complex			
	Yes	No	*χ2* ^†^	*p-*value
	n (%)	n (%)		
**Sex**				
Male	212 (51.6)	199 (48.4)	5.394	0.020*
Female	126 (61.5)	79 (38.5)		
**Age**				
18–39 years	150 (55.8)	119 (44.2)	1.540	0.463
40–59 years	144 (52.6)	130 (47.4)		
≥60 years	44 (60.3)	29 (39.7)		
**Marital status**				
Single	44(48.9)	46 (51.1)	1.523	0.217
Ever married	294(55.9)	232 (44.1)		
**Education**				
Primary	5(62.5)	3 (37.5)	1.630	0.653
Secondary	18 (52.9)	16 (47.1)		
Higher secondary	112 (58.3)	80 (41.7)		
Bachelor’s degree or higher	203 (53.1)	179 (46.9)		
**Occupation**				
Job holder	144 (52.4)	131 (47.6)	10.277	0.036*
Business	50 (51.0)	48 (49.0)		
Student	28 (47.5)	31 (52.5)		
Retired	32 (54.2)	27 (45.8)		
Housewife	84 (67.2)	41 (32.8)		
**Religion**				
Muslim	313 (55.6)	250 (44.4)	3.731	0.155
Hindu	23 (45.1)	28 (54.9)		
Buddhist	2 (100.0)	0 (0.0%)		
**Monthly family income**				
<50,000 BDT	130 (60.2)	86 (39.8)	3.995	0.136
50,000–99,000 BDT	143 (52.8)	128 (47.2)		
≥100,00 BDT	65 (50.4)	64 (49.6)		
**Smoking status**				
Smoker	85 (70.2)	36 (29.8)	14.380	<0.001***
Non-smoker	253 (51.1)	242 (48.9)		
**Smoking by family members**				
Yes	102 (75.0)	34 (25.0)	28.560	<0.001***
No	236 (49.2)	244 (50.8)		
**Length of stay at home**				
≤12 hours	166(50.6)	162 (49.4)	5.142	0.023*
>12 hours	172(59.7)	116 (40.3)		
**Type of housing complex**				
Private	252 (53.5)	219 (46.5)	1.510	0.219
Government	86 (59.3)	59 (40.7)		
**Place of residence**				
Dhaka city	135(63.1)	79 (36.9)	15.615	0.016*
Chattogram city	36(53.7)	31 (46.3)		
Rajshahi city	34(50.7)	33 (49.3)		
Khulna city	39(58.2)	28 (41.8)		
Sylhet city	25(37.3)	42 (62.7)		
Barishal city	34(50.7)	33 (49.3)		
Rangpur city	35(52.2)	32 (47.8)		

Notes

^†^Pearson’s chi-square

*p < 0.05

***p < 0.001

### Predictors of SHS exposure within MUH complexes

The results of multivariable logistic regression analysis are provided in Table 3. By the sex category, females were 1.8 times more likely to be exposed to SHS within MUH complexes than their male counterparts (95% CI = 1.236–2.681). The respondents with a monthly family income of below 50,000 BDT had a 1.9 times higher chance of being exposed to SHS within MUH complexes than those having a monthly family income of 100,000 BDT or more (95% CI = 1.162–3.220). Smokers were 2.6 times more likely to be exposed to SHS within MUH complexes than their non-smoking counterparts (95% CI = 1.643–4.252). The respondents whose family members were smokers had a 2.4 times higher chance of being exposed to SHS within MUH complexes compared to those whose family members were not smokers (95% CI = 1.537–3.746). Furthermore, the residents of Dhaka city were at 1.9 times higher risk of experiencing SHS exposure within MUH complexes than those from Rangpur city (95% CI = 1.013–3.440).

**Table 3:** Factors associated with SHS exposure among MUH residents.

Variable	Category	aOR	95% CI	*p*-value
**Sex**	Female	1.8	1.236–2.681	0.002**
	Male	Reference		
**Monthly family income**	<50,000 BDT	1.9	1.162–3.220	0.011*
	50,000–99,000 BDT	1.4	0.868–2.228	0.171
	≥100,000 BDT	Reference		
**Smoking status**	Smoker	2.6	1.643–4.252	<0.001***
	Non-smoker	Reference		
**Smoking by family members**	Yes	2.4	1.537–3.746	<0.001***
	No	Reference		
**Place of residence**	Dhaka city	1.9	1.013–3.440	0.045*
	Chattogram city	1.3	0.644–2.741	0.442
	Rajshahi city	1.1	0.517–2.136	0.890
	Khulna city	1.3	0.645–2.699	0.447
	Sylhet city	0.7	0.342–1.470	0.355
	Barishal city	0.9	0.424–1.761	0.687
	Rangpur city	Reference		

*Notes*: aOR = Adjusted Odds Ratio; CI = Confidence Interval

*p < 0.05

**p < 0.01

***p < 0.001

## Discussion

We carried out this study to investigate the prevalence of SHS exposure and its associated factors among MUH residents in the seven Bangladeshi divisional cities. Over half (54.9%) of the respondents living in MUH where there was no smoke-free policy were exposed to SHS within MUH complexes during the last month. This prevalence of SHS exposure is higher than that reported in other studies. A study conducted in New York State showed that 46.2% of residents living in MUH with a smoke-free home policy experienced SHS incursions in their units from somewhere else in or around their buildings during the last 12 months [26]. Another US study found that 49.9% of MUH residents who followed smoke-free home rules reported SHS incursions into their units, which originated in neighbouring units, common areas, or adjacent buildings during the last 12 months [27]. The higher prevalence of SHS exposure in Bangladesh could be mainly due to the absence of smoke-free home policies in MUH complexes. However, the findings suggest the need to implement a comprehensive smoke-free policy in MUH complexes to fully protect residents from exposure to SHS. In adherence to the tobacco control legislation, Bangladesh has implemented prohibitions on smoking in numerous public venues and on public transportation. Given the significant extent of SHS exposure, it is within the purview of the government to potentially enact legal measures to prohibit tobacco smoking within MUH complexes. Moreover, the government’s dedication to establishing a tobacco-free Bangladesh by the year 2040 possesses the capacity to guarantee the existence of smoke-free surroundings within residential areas, which serves as a significant contributor to SHS exposure among individuals who do not engage in smoking activities.

In the present study, females were more likely to be exposed to SHS within housing complexes than males. This finding is supported by prior research conducted in Bangladesh and other Asian countries [28–30]. The majority of Bangladeshi females are housewives who spend most of their time inside housing complexes, particularly at home. Due to their prolonged stay inside complexes, they may have been at increased risk of SHS exposure at home and from neighbouring units, common areas, and adjacent buildings.

The present study revealed that the respondents from a low-income group had a higher risk of experiencing SHS exposure within MUH complexes compared to those from a high-income group. Similar findings have been observed in previous studies conducted in China, Kuwait, and the United States of America [31–33]. One possible explanation for this higher prevalence of SHS exposure is that households with a low income live in small flats where one room is attached to another so closely that non-smoking family members are easily exposed to tobacco smoke when other members smoke inside flats [34, 35].

The present study showed that smoking residents had higher odds of being exposed to SHS within MUH complexes than their non-smoking counterparts. This finding is congruent with previous studies conducted in Malaysia and Portugal [36, 37]. The higher prevalence of SHS exposure can be explained by the fact that residents who smoke are more likely to have friendships with smoking residents compared to non-smoking residents, and therefore have a higher risk of being exposed to SHS [38]. Furthermore, smokers are less likely to perceive SHS as harmful than non-smokers, and this may have discouraged them to avoid SHS exposure [39].

According to the results of the present study, MUH residents whose family members smoked were more likely to be exposed to SHS within housing complexes than those whose family members did not smoke. This finding is consistent with several earlier investigations done in Bangladesh and other countries [28, 40, 41]. Due to lack of smoking restrictions, many people smoke in their homes, which leads to increased exposure to SHS among non-smoking family members [42].

Among the respondents, those living in Dhaka city had the highest exposure to SHS within housing complexes. Evidence shows that a large portion of land owners in Dhaka city do not maintain an adequate distance from one building to another [43, 44]. Because of the shorter inter-building distance, MUH residents in Dhaka city may have experienced more SHS incursions from adjacent buildings than those in other cities in Bangladesh.

### Limitations

This research has a number of drawbacks. First, due to the cross-sectional design, causal relationships between the outcome variable and the independent variables could not be established in this study. Second, all data were self-reported by the participants, which could have led to recollection and social desirability bias. Third, SHS exposure was evaluated based on the ability to smell SHS. This evaluation may have led to disparities between perceived and true SHS exposure levels. Fourth, this study recruited more male respondents than female ones, which may have biased the results. In the cultural context of Bangladesh, it is commonly observed that women often experience a sense of embarrassment when engaging in conversation with unknown individuals, particularly those of the male gender. Another concern, namely that of security, might have deterred women from independently opening doors when their male or elderly family members were not present at home. Hence, more male residents agreed to participate in the interviews than their female counterparts. Finally, we did not collect data regarding the number of units within sampled MUH complexes. The quantity of SHS exposure frequently varies depending on the number of units in a complex; thus, we recognize that it could be crucial to determine the number of units inside sampled complexes. In a complex with more units, there are more residents. Similar to this, a complex with more occupants will likely have more smokers. The likelihood that smokers will smoke in communal spaces increases with the number of smokers in the complex, putting non-smokers at increased risk of SHS exposure. We were unable to demonstrate how the number of units in a MUH complex affected the level of SHS exposure among non-smoking inhabitants due to lack of information on the number of units.

## Conclusions

The present study revealed a high prevalence of SHS exposure among MUH residents living in the divisional cities of Bangladesh. SHS exposure was significantly related with females, low income groups, smokers, those with smoking family members, and Dhaka city residents. The findings indicate an urgent need to minimize SHS exposure among MUH residents across the country. To protect non-smoking residents from SHS exposure, the Government of Bangladesh should adopt a smoking ban in and around housing complexes through revisions in the current tobacco control law and the national housing policy. Furthermore, cessation services, such as behavioural counselling and pharmacotherapy can be introduced in MUH complexes so that smokers are able to quit smoking quickly.

## Supporting information

S1 File Study protocol. (DOCX)

S2 File Questionnaire. (DOCX)

S3 File Raw dataset. (XLSX)

S4 File Codebook. (DOCX)

## References

[1] Öberg M, Woodward A, Jaakkola MS, Peruga A, Prüss-Üstün A. Global estimate of disease from second-hand smoke. Geneva: World Health Organization; 2010.

[2] Centers for Disease Control and Prevention (CDC). Tobacco [Internet]. 2017 (cited 2022 Apr 04). Available from: https://www.cdc.gov/biomonitoring/tobacco.html

[3] US Department of Health and Human Services. The health consequences of involuntary exposure to tobacco smoke: a report of the Surgeon General. Atlanta, GA: US Department of Health and Human Services, Centers for Disease Control and Prevention, Coordinating Center for Health Promotion, National Center for Chronic Disease Prevention and Health Promotion, Office on Smoking and Health, 2006.

[4] He J, Vupputuri S, Allen K, et al. Passive smoking and the risk of coronary heart disease-a meta-analysis of epidemiological studies. *N Engl J Med*. 1999, 340:920–926. doi: 10.1056/NEJM199903253401204 10089185

[5] Oono IP, Mackay DF, Pell JP. Meta-analysis of the association between secondhand smoke exposure and stroke. *Journal of Public Health*. 2011;33(4):496–502. doi: 10.1093/pubmed/fdr025 21422014

[6] World Health Organization. Women’s exposure to second-hand smoke: a serious health concern [Internet]. 2012 (cited 2022 Jun 05). Available from: https://www.who.int/china/news/detail/06-11-2012-women-s-exposure-to-second-hand-smoke-a-serious-health-concern

[7] National Center for Chronic Disease Prevention and Health Promotion (US) Office on Smoking and Health. The Health Consequences of Smoking—50 Years of Progress: A Report of the Surgeon General. Atlanta (GA): Centers for Disease Control and Prevention (US); 2014. 24455788

[8] Jinot J, Bayard S. Respiratory health effects of exposure to environmental tobacco smoke. *Rev Environ Health*. 1996;11:89–100. doi: 10.1515/reveh.1996.11.3.89 9000301

[9] Fact sheet: Tobacco [Internet]. Geneva: World Health Organization; 2022 May 24. Available from: https://www.who.int/news-room/fact-sheets/detail/tobacco

[10] Bangladesh Bureau of Statistics and National Tobacco Control Cell. Global adult tobacco survey Bangladesh 2017. Dhaka: BBS and NTCC; 2019

[11] Faruque GM, Ahmed M, Huq I, Parven R, Wadood SN, Chowdhury SR, et al. The economic cost of tobacco use in Bangladesh: A health cost approach [Internet]. Dhaka: Bangladesh Cancer Society; 2020 (cited 2023 Jan 12). Available from: http://bnttp.net/wp-content/uploads/2020/04/Bangladesh-Health-Cost-Full-Report-2020.pdf

[12] UNCTAD. Total and urban population [Internet]. Geneva: UNCTAD; 2022 (cited 2023 Jan 15). Available from: https://hbs.unctad.org/total-and-urban-population/ https://hbs.unctad.org/total-and-urban-population/.

[13] Lathen LS, Plears ML, Shartle EL, Conner KL, Fiore MC, Christiansen BA. The HUD smoke-free rule: Perceptions of residents post-implementation. *Prev Med Rep*. 2020;19:101159. doi: 10.1016/j.pmedr.2020.101159 32728524 PMC7381686

[14] Kaufman P, Kang J, Kennedy RD, Beck P, Ferrence R. Impact of smoke-free housing policy lease exemptions on compliance, enforcement and smoking behavior: A qualitative study. *Preventive Medicine Reports*.2018;10, 29–36. doi: 10.1016/j.pmedr.2018.01.011 29552455 PMC5852412

[15] Grace C, Greenhalgh EM, Tumini V. 15.6 Smoking bans in the home and car. In: Greenhalgh EM, Scollo MM, Winstanley MH, editors. Tobacco in Australia: Facts and issues. Melbourne: Cancer Council Victoria; 2022 (cited 2023 Jan 15). Available from: http://www.tobaccoinaustralia.org.au/chapter-15-smokefree-environment/15-6-domestic-environments

[16] Pizacani BA, Maher JE, Rohde K, Drach L, Stark MJ. Implementation of a smoke-free policy in subsidized multiunit housing: effects on smoking cessation and secondhand smoke exposure. Nicotine Tob Res. 2012;14(9), 1027–1034. doi: 10.1093/ntr/ntr334 22318686

[17] Smoking and Tobacco Products Usage (Control) Act, 2005 [Internet], 2005 Mar 15 (cited 2022 Jun 11) (Bangladesh). Available from: https://www.banglajol.info/index.php/SSR/article/view/56516/39441

[18] National Housing Authority. National housing policy 2016. Available from: http://nha.portal.gov.bd/sites/default/files/files/nha.portal.gov.bd/law/76f125dc_8e5e_4095_b03d_7d9ac29f842d/National%20Housing%20Policy%202016_English%20Version.pdf

[19] Schoenmarklin S. Secondhand smoke seepage into multi-unit affordable housing [Internet]. St. Paul (MN): Tobacco Control Legal Consortium; 2010 (cited 2023 Jan 15). Available from: https://publichealthlawcenter.org/sites/default/files/resources/tclc-syn-secondhand-2010.pdf

[20] Wilson KM, Klein JD, Blumkin AK, Gottlieb M, Winickoff JP. Tobacco-smoke exposure in children who live in multiunit housing. *Pediatrics*. 2010;127(1):85–92. doi: 10.1542/peds.2010-2046 21149434

[21] World Health Organization, Regional Office for South-East Asia. Global Youth Tobacco Survey (GYTS): Bangladesh report, 2013. New Delhi: WHO-SEARO, 2015

[22] Jallow IK, Britton J, Langley T. Prevalence and factors associated with exposure to secondhand smoke (SHS) among young people: a cross-sectional study from the Gambia. *BMJ Open*. 2018;8(3):e019524. doi: 10.1136/bmjopen-2017-019524 29540414 PMC5857680

[23] Cochran WG. Sampling techniques. 2nd ed. New York: John Wiley and Sons, Inc.; 1963

[24] Tsang S, Royse CF, Terkawi AS. Guidelines for developing, translating, and validating a questionnaire in perioperative and pain medicine. *Saudi J Anaesth*. 2017;11(Suppl 1):S80–S89. doi: 10.4103/sja.SJA_203_17 28616007 PMC5463570

[25] Chan YH. Biostatistics 202: logistic regression analysis. *Singapore Med J*. 2004;45(4):149–153. 15094982

[26] King BA, Cummings KM, Mahoney MC, Juster HR, Hyland AJ. Multiunit housing residents’ experiences and attitudes toward smoke-free policies. *Nicotine Tob Res*. 2010:12(6):598–605. doi: 10.1093/ntr/ntq053 20395360 PMC3436441

[27] Gentzke AS, Hyland A, Kiviniemi M, Travers MJ. Attitudes and experiences with secondhand smoke and smoke-free policies among subsidised and market-rate multiunit housing residents living in six diverse communities in the USA. *Tob Control*. 2018;27(2):194–202. doi: 10.1136/tobaccocontrol-2016-053374 28302920 PMC5844180

[28] Abdullah AS, Driezen P, Sansone G, Nargis N, Hossain GA, Quah AC, et al. Correlates of exposure to secondhand smoke (SHS) at home among nonsmoking adults in Bangladesh: findings from the ITC Bangladesh survey. *BMC Pulm Med*. 2014;14(1):117. doi: 10.1186/1471-2466-14-117 25027238 PMC4107590

[29] Verma M, Kathirvel S, Das M, Aggarwal R, Goel S. Trends and patterns of second-hand smoke exposure amongst the non-smokers in India-A secondary data analysis from the Global Adult Tobacco Survey (GATS) I & II. *Plos ONE*. 2020; 15(6): e0233861. doi: 10.1371/journal.pone.0233861 32520979 PMC7286505

[30] Wang CP, Ma SJ, Xu XF, Wang JF, Mei CZ, Yang GH. The prevalence of household second-hand smoke exposure and its correlated factors in six counties of China. *Tob Control*. 2009;18(2), 121–126. doi: 10.1136/tc.2008.024836 19131456 PMC2655043

[31] Yang Y, Yang XY, Yang T, He W, Peng S, Rockett IR. Social deprivation and secondhand smoke exposure among urban male residents: A nationwide study in China. *Tob Induc Dis*. 2021;19:21. doi: 10.18332/tid/132290 33767605 PMC7983223

[32] Ziyab AH, Almari M, Al-Taiar A. Exposure to household secondhand smoke among adolescents in Kuwait: Results from two school-based cross-sectional studies. *Tob Induc Dis*. 2020;18:32. doi: 10.18332/tid/119116 32336970 PMC7177388

[33] Wilson KM, Torok M, McMillen R, Tanski S, Klein JD, Winickoff JP. Tobacco smoke incursions in multiunit housing. *Am J Public Health*. 2014;104(8):1445–53. doi: 10.2105/AJPH.2014.301878 24922124 PMC4103233

[34] Rowa-Dewar N, Amos A, Cunningham-Burley S. Children’s perspectives on how parents protect them from secondhand smoke in their homes and cars in socioeconomically contrasting communities: a qualitative study. *Nicotine Tob Res*. 2014;16(11):1429–1435. doi: 10.1093/ntr/ntu096 24951494

[35] Kim J, Lee K, Kim K. Factors associated with secondhand smoke incursion into the homes of non-smoking residents in a multi-unit housing complex: a cross-sectional study in Seoul, Korea. *BMC Public Health*. 2017;17(1):739. doi: 10.1186/s12889-017-4774-x 28946863 PMC5613333

[36] Ling MYJ, Lim KH, Hasani WSR, Rifin HM, Majid NLA, Lourdes TGR, et al. Exposure to secondhand smoke among school-going adolescents in Malaysia: Findings from the tobacco and e-cigarettes survey among Malaysian adolescents (TECMA). *Tob Induc Dis*. 2020;18:96. doi: 10.18332/tid/128622 33262682 PMC7694740

[37] Alves RF, Precioso J, Becoña E. Smoking behavior and secondhand smoke exposure among university students in northern Portugal: Relations with knowledge on tobacco use and attitudes toward smoking. *Pulmonology*. 2020;28(3):193–202. doi: 10.1016/j.pulmoe.2020.03.004 32444313

[38] Schaefer DR, Haas SA, Bishop NJ. A dynamic model of US adolescents’ smoking and friendship networks. *Am J Public Health*. 2012;102(6):e12–e18. doi: 10.2105/AJPH.2012.300705 22515861 PMC3349762

[39] King BA, Dube SR, Babb SD. Perceptions about the harm of secondhand smoke exposure among U.S. middle and high school students: findings from the 2012 National Youth Tobacco Survey. *Tob Induc Dis*. 2013;11(1):16. doi: 10.1186/1617-9625-11-16 23867000 PMC3717105

[40] Lim HL, Teh CH, Kee CC, Ghazali SM, Pan SA, Lim KH. Exposure to second-hand smoke among secondary school-going adolescents: Findings from the Malaysian Adolescent Health Risk Behaviour (MyAHRB) study. *Proc Singapore Healthc*. 2019:19–25. doi: 10.1177/2010105818789961

[41] Phetphum C, Noosorn N. Prevalence of secondhand smoke exposure at home and associated factors among middle school students in Northern Thailand. *Tob Induc Dis*. 2020;18:11. doi: 10.18332/tid/117733 32165877 PMC7057047

[42] Desalu OO, Onyedum CC, Adewole OO, Fawibe AE, Salami AK. Secondhand smoke exposure among nonsmoking adults in two Nigerian cities. *Ann Afr Med*. 2011;10(2):103–111. doi: 10.4103/1596-3519.82069 21691015

[43] Hossain MM. Rajuk and developers. The Daily Star. 2010 Jun 10 [cited 2022 Jul 17]. Available from: https://www.thedailystar.net/news-detail-143452

[44] Karim R. Changing designs, flouting codes. The Business Standard. 2022 Feb 26 [cited 2023 Jan 24]. Available from: https://www.tbsnews.net/bangladesh/infrastructure/changing-designs-flouting-codes-376357

