# Supplementary material for: Secondhand smoke exposure and associated factors among city residents living in multiunit housing in Bangladesh
Source: PLoS One. 2023 Sep 21;18(9):e0291746. doi: 10.1371/journal.pone.0291746 (PMC10513191; doi:10.1371/journal.pone.0291746)
Supplement: S1 File — (DOCX) [file pone.0291746.s001.docx]

**Study protocol**

**Secondhand smoke exposure and associated factors among city residents living in multiunit housing in Bangladesh**

**1.0 Introduction**

**1.1 Background:**

Secondhand smoke (SHS) exposure is a major public health issue worldwide. According to the World Health Organization (WHO), SHS is formed from the burning of cigarettes and other tobacco products and from smoke exhaled by the smoker [1]. SHS is the combination of smoke from the burning end of a cigarette and the smoke breathed out by smokers [2]. SHS exposure is expressed as the sum of exposures in the multiple microenvironments where a person spends time [3]. The exposure mainly consists of the smoke released from the burning end of a smoldering cigarette, pipe, or cigar and, to a lesser extent, the smoke exhaled from the lungs of an active smoker nearby [4].

The population of Bangladesh is highly susceptible to SHS exposure because of high smoking rates and low awareness about the harmful effects of SHS. Globally, more than 7 million deaths occur due to tobacco use annually, while more than 600,000 are the result of exposure to SHS [5]. In Bangladesh, 35.3% of adults (37.8 million) currently use tobacco in smoking or smokeless form and 39.0% are exposed to SHS exposure at home [6]. Every year more than 57,000 people die, and 1.2 million people suffer from diseases in Bangladesh due to tobacco use; 16% of all deaths among people aged 30 years and above are attributable to tobacco use [7].

Multiunit housing (MUH) is a popular housing concept in urban Bangladesh. Urbanization is proceeding rapidly in the country as every year thousands of people migrate to cities and towns to earn a better livelihood. In 1991, about 22.0% of the total population lived in urban areas, whereas 36.6% lived in urban areas in 2018. It is estimated that by 2046, more than 50% of the country’s total population will live in urban areas [8]. The rate of urbanization is much faster in cities than towns. With an increase in the urban population, the amount of land is not increasing. In order to meet the housing needs of this increasing population, MUH complexes that can accommodate many families at a time are mushrooming in these cities. People living in MUH complexes are susceptible to SHS exposure because tobacco smoke seeps into their flats through windows, doorways, ventilation systems, electrical outlets, etc. from adjacent flats and even outdoors or they are directly exposed to SHS in common spaces. A study done in a sample of 1,030 MUH residents in New York City revealed that two-thirds (67.4%) of the respondents reported seeing people smoke in common areas during the past 12 months and on average nicotine concentrations were higher in New York City Housing Authority (NYCHA) apartments and hallways than government subsidized housing [9]. A study done in Minnesota by Hewett et al revealed that 48.0% of the residents from MUH complexes with a smoking ban in only flats smelled tobacco smoke at times in their flats, which originated elsewhere in or around the building [10]. Findings from a study in Denmark showed that among the respondents living in multiunit dwellings who never allowed smoking in their flats, 28.2% were exposed to neighbour smoke [11]. As reported by a study done in Seoul, South Korea, 44.2% of nonsmoking residents experienced SHS incursions into their home once a week or more [12]. A study among never-smoking adolescents in Hongkong reported that one-third of the respondents (33.2%) experienced SHS exposure at home, including 16.2% from inside the home, 10.0% from neighbours and 7.0% from both [13].

The Government of Bangladesh has undertaken some initiatives for tobacco control. In 2004, Bangladesh ratified the WHO Framework on Convention on Tobacco Control (FCTC). Following the convention, the country enacted the ‘Smoking and Tobacco Products Usage (Control) Act’ in 2005 [14]. The Act was further strengthened through an Amendment in 2013.The Government of Bangladesh aims to work towards full compliance with the FCTC to make the country tobacco free by 2040 [15]. Article 8 of the FCTC warrants protection from exposure to tobacco smoke [16], which suggests the total elimination of smoking and tobacco smoke in a particular environment. The Act has declared 24 categories of places as public places and 8 categories of transports as public transports where smoking is completely banned.

MUH residents are at greater risk of SHS exposure, but this issue still remains overlooked. Until today, there is not a single study done to determine the prevalence of SHS exposure among MUH residents in Bangladesh. Therefore, this study aims to assess the level of SHS exposure and identify the associated factors among people living in MUH complexes in the divisional cities of Bangladesh.

**1.2 Justification of the study**

A sizeable portion of urban dwellers live in MUH complexes in the divisional cities of Bangladesh. There are some common spaces within the complex, and these include waiting spaces, parking areas, basements, stairs, roofs, doorways and entrances of the complex. These spaces are shared by all residents of the complex. When smoker residents smoke in these common spaces, other residents standing or moving there are exposed to the smoke, and sometimes this smoke penetrates into flats in the same housing complex and other housing complexes as well, causing residents exposed to SHS. Though the existing tobacco control law has prohibited smoking in some selected public places and on public transports, it has not included these common spaces as a public place. Furthermore, MUH residents are exposed to tobacco smoke in their own flats. Importantly, there is a separate housing policy in the country, called “National Housing Policy” which has not restricted tobacco use in the housing setting [17]. Due to the absence of tobacco control provisions in the tobacco control law and the housing policy, residents are free to smoke tobacco inside the housing complex and somewhere else close to it, which eventually heightens non-smoking residents’ vulnerability to involuntary SHS exposure. Modifications in the tobacco control law and/or the housing policy with a focus on tobacco restrictions in housing or developing a comprehensive smoke-free housing policy can protect MUH residents from SHS exposure. Therefore, scientific evidence on the prevalence and magnitude of secondhand smoke exposure among housing residents is needed for upgrading the tobacco control law and/or the housing policy or developing a comprehensive smoke-free housing policy.

Exposure to SHS is a preventable cause of morbidity and mortality. Tobacco smoke contains more than 7,000 chemical components, and at least 250 of these chemicals are detrimental to human health [18]. There is no risk-free level of exposure to SHS, and even short-term exposure can contribute to disease pathogenesis [19,20]**.** Involuntary smoking increases the risk of cardiovascular diseases (CVDs). Evidence shows that SHS exposure is a risk factor for heart disease [21,22] and stroke [21,23] which are respectively the largest and the second largest cause of death worldwide. SHS exposure is also a major risk factor for lung cancer among nonsmokers. Nonsmokers who are exposed to SHS are 25-30% more likely to develop lung cancer compared to those who are not exposed to SHS [24]. Children and women are most exposed to involuntary smoking because they stay at home for a longer time than male adults. SHS exposure contributes to the development of bronchitis, pneumonia, asthma, ear infections, neurodevelopmental problems and sudden infant death syndrome (SIDS) in children [21,25]. SHS exposure affects the reproductive health of women. According to the World Health Organization (WHO), women of reproductive age experience adverse reproductive health outcomes, such as pregnancy complications, fetal growth restriction, preterm delivery, stillbirths, and infant death due to tobacco use and SHS exposure [26]. However, these SHS-related health problems among housing residents can be reduced if they are protected from the exposure to SHS in MUH complexes, especially common spaces.

**2.0 Objectives of the study**

**2.1 General objective**

To assess the exposure of secondhand smoke and identify its associated factors among city residents living in multiunit housing in the divisional cities of Bangladesh

**2.2 Specific Objectives**

- To assess the prevalence of secondhand smoke exposure among city residents from common spaces within the multiunit housing complex
- To determine the prevalence of secondhand smoke exposure among city residents in their flats within the multiunit housing complex
- To assess the prevalence of secondhand smoke exposure among city residents living in multiunit housing from next flats
- To measure the prevalence of secondhand smoke exposure among city residents living in multiunit housing from next buildings
- To find out the frequency of secondhand smoke exposure within the multiunit housing complex among city residents
- To identify associations between basic characteristics and secondhand smoke exposure among city residents living in multiunit housing

**3.0 Variables of the study**

**3.1 Outcome variable**

Exposure to secondhand smoke within the MUH complex among city residents will be the outcome variable for this study. SHS exposure will be assessed using the question, “On how many days during the past 30 days, did you get the smell of cigarettes/bidis from the following places: (a) your own flats, (b) next flats, (c) common spaces and (d) next buildings?” Those who reported any number between 1 and 30 days were considered exposed to SHS from the particular place [27]. Overall SHS exposure within the MUH complex will be defined as any exposure from own flats, next flats, common spaces and next buildings during the past 30 days.

**3.2 Independent variables**

**Sociodemographic variables:**

This study will include the following basic information-related variables: sex, age (in years), marital status, education level, occupation, monthly family income, length of stay at home (in terms of hours per day), type of housing complex (private or government), flat ownership (owned or rented), place of residence (Dhaka city, Chattogram city, Rajshahi city, Khulna city, Sylhet city, Barishal city or Rangpur city).

**Smoking status-related variables:**

Respondents will be asked about their smoking status (smoker or non-smoker). Their smoking status will be determined by asking, “On how many days during the past 30 days, did you smoke cigarettes/bidis?” Those who smoked cigarettes/bidis on at least one day during the past 30 days will be categorized as ‘smokers’, and those who did not smoke cigarettes/bidis during the past 30 days will be categorized as ‘non-smokers’ [28]. Furthermore, respondents will be asked whether there are smokers in their family (Yes or No).

**4.0 Methodology**

**4.1 Study design**

This study will follow a cross-sectional design with a quantitative approach.

**4.2 Study population**

The target population includes all adult residents of MUH complexes with a management committee in the divisional cities of Bangladesh.

**4.3 Study sites**

This study will be conducted in seven (07) divisional cities in Bangladesh – Dhaka, Chattogram, Khulna, Rajshahi, Sylhet, Rangpur and Barishal. In this study, Mymensingh will be considered part of Dhaka division.

**4.4 Study period**

This study will be conducted from April 2019 to November 2019.

**4.5 (a) Sample size calculation**

The minimum required sample size will be calculated using a single population proportion formula:

n = (z^2^pq/e^2^)

Where,

n = Desired sample size

z = Standard normal deviate = 1.96 at 95% confidence interval

p = Prevalence (unknown) of secondhand smoke exposure among city residents living in multiunit housing (unknown) = 50% = 0.50

q = 1- p =1-0.50 = 0.50

e = Margin of error = 4% =0.04

n = (1.96^2^*0.50*0.50)/0.04^2^

= **600**

**(b) Sample Size Distribution**

There is no list of MUH complexes across the seven divisional cities of Bangladesh. That’s why, I will prepare a list of MUH complexes in these cities. The number of MUH complexes in Dhaka city is much higher than that in each of other six cities. Dhaka city’s number of MUH complexes being very large, I will enlist 1,000 MUH complexes from this city, whereas I will enlist all MUH complexes from other six cities. Therefore, I have decided to assign about one-third (35%) sample to Dhaka city and distribute 65% sample among other six cities. Furthermore, I will interview maximum five (5) respondents from each MUH complex. The sample distribution of this study is shown in the following table:

| Sl # | City | % of total sample size | Sample | Minimum no. of MUH complexes |
| --- | --- | --- | --- | --- |
| 1 | Dhaka | 35% | 210 | 42 |
| 2 | Chattogram | 11% | 65 | 13 |
| 3 | Sylhet | 11% | 65 | 13 |
| 4 | Khulna | 11% | 65 | 13 |
| 5 | Rajshahi | 11% | 65 | 13 |
| 6 | Rangpur | 11% | 65 | 13 |
| 7 | Barishal | 11% | 65 | 13 |
|  | **Total** | **100%** | **600** | **120** |

**4.6 Inclusion criteria**

- Residents aged 18 years and above
- Residents who have been living in MUH complexes for at least 2 years
- Those who will be available at home during data collection

**4.7 Exclusion criteria**

- Residents having speech and/or hearing impairments
- Pregnant women
- Those who will not agree to participate in the interview

**4.8 Sampling technique**

First, we will select a total of minimum 121 MUH complexes (42 from Dhaka city and 13 from each of the six divisional cities *randomly* using the newly prepared list of MUH complexes. Second, we will select 600 respondents at the household level from the selected MUH complexes *conveniently*.

**5.0 Data collection tools**

A semi-structured questionnaire will be used for this study. The questionnaire will contain the respondents’ information on (i) basic information: age, sex, education, marital status, occupation, religion, monthly family income, type of MUH complex and distance complexes (ii) smoking behaviours: smoking status, type of smokers, age of smoking initiation, total duration of smoking and no. of smokers (iii) knowledge, attitudes and perceptions (KAP) about SHS exposure**:** knowledge about health effects of SHS exposure and perceptions about SHS exposure and (iv) frequency, pattern and magnitude of SHS exposure: length of stay at home, SHS exposure in common spaces during last 30 days, SHS exposure in own flat during last 30 days, SHS exposure from next flat during last 30 days, SHS exposure from next building during last 30 days, frequency of SHS exposure per day and length of SHS exposure per day.

We will use the Bengali version questionnaire for data collection. The English version questionnaire will be first translated into Bengali and then back translated into English for ensuring the quality and accuracy of the content. However, the questionnaire will be field tested before the formal starting of data collection.

**6.0 Data analysis plan**

Data will be analyzed using SPSS software version 25. The continuous variables will be expressed as mean and standard deviation (SD), and the categorical variables will be expressed as frequencies (%). Bivariate analyses will be performed by t-test and chi-square test for the continuous variables and categorical variables, respectively. The logistic regression model will be used to identify predictors of SHS exposure. The associations between the independent variables and outcome variable will be presented as adjusted odds ratios (ORs) and 95% confidence interval (CI). All statistics will be tested using a two-sided test, and a p value of <0.05 will be considered statistically significant.

**7.0 Quality control**

Data collection staff members, including Data Collectors, Qualitative Interviewers and Field Supervisors were provided with a 3-day training on data collection. The training was designed in such a manner that the data collection staff got a thorough understanding of the study objectives, study methodology, including sample design, location, identification of the sample, etc. They were taught the techniques of rapport building, environment control, maintenance of objectivity and neutrality, techniques of conducting a face-to-face interview, techniques of avoiding biases in asking questions, techniques of handling difficult respondents and techniques of getting respondents’ consent before the start of the interview. Principal Investigator and Co-Investigator jointly provided this training. The training was conducted in classroom lecture, demonstration interview, mock interview, front-of-class interview, review of lessons learnt and field practice interview approaches. Following the field practice interview session, classroom discussions took place so that the participants could get a clear understanding of the questionnaire and remove confusions. For ensuring the quality of data collected, Field Supervisors spot checked at least 20 % of the interviews randomly. Principal Investigator and Co-Investigator took turns throughout data collection to make on-site field checks.

**8.0 Ethical considerations**

Ethical approval for this study will be taken from the national research ethics body *Bangladesh Medical Research Council (BMRC)* before the conduct of this study. Respondents’ participation in the study is completely voluntary. Informed consent will be obtained from the respondents. The respondents will be assured that the information shared will remain confidential and will not be used for any purpose other than the study. Data collection staff members will be trained to treat all respondents with respect, to be fair and honest, and to avoid applying any pressure on respondents. To protect individuals’ confidentiality following their participation in the research, each respondent’s name will be paired with a code number. However, if any respondent does not agree to participate in the study, he/she will not be interviewed.

**9.0 Expected outcomes**

To date, no research has been conducted in Bangladesh to assess the prevalence of secondhand smoke exposure in MUH complexes. The results of this study may help policymakers adopt a new policy or modify existing policies to ban smoking in MUH complexes.

The results of this study can be of great use to tobacco control professionals/activists. They can design new interventions that may reduce tobacco use in MUH complexes and help smokers quit smoking.

The results of this study can be used baseline data for other researchers. Using the results, they can conduct more research on secondhand smoke exposure that will contribute to further strengthening tobacco control measures throughout the country.

**References**

1. Öberg M, Woodward A, Jaakkola MS, Peruga A, Prüss-Üstün A. Global estimate of disease from second-hand smoke*.* Geneva: World Health Organization; 2010.

2. U.S. Department of Health and Human Services. The health consequences of involuntary exposure to tobacco smoke: A report of the Surgeon General. Atlanta, GA: U.S. Department of Health and Human Services, Centers for Disease Control and Prevention, National Center for Chronic Disease Prevention and Health Promotion, Office on Smoking and Health, 2006.

3. Avila-Tang E, Elf JL, Cummings KM, Fong GT, Hovell MF, Klein JD, McMillen R, Winickoff JP, Samet JM. Assessing secondhand smoke exposure with reported measures. *Tob Control*. 2013; 22(3):156–63.

4. Dunbar A, Gotsis W, Frishman W. Second-hand tobacco smoke and cardiovascular disease risk: an epidemiological review. *Cardiol Rev*. 2013; 21:94–100.

5. World Health Organization. WHO Report on the Global Tobacco Epidemic, 2017: Monitoring tobacco use and prevention policies [Internet]. 2017 (cited 2022 Jun 14). Available from: <https://www.who.int/publications/i/item/9789241512824>

6. Bangladesh Bureau of Statistics. Preliminary report on global adult tobacco survey (GATS), Bangladesh 2017 [Internet]. 2018 (cited 2022 Jun 08). Available from: <http://bbs.portal.gov.bd/sites/default/files/files/bbs.portal.gov.bd/page/57def76a_aa3c_46e3_9f80_53732eb94a83/Preliminary%20Report%20on%20GATS%20Bangladesh%202017.pdf>

7. World Health Organization. Impact of tobacco-related illnesses in Bangladesh. New Delhi: World Health Organization Regional Office for South-East Asia; 2007.

8. IUCN and BFD. Bangladesh National Conservation Strategy, Part II: Sectoral Profile. Dhaka: IUCN; 2016.

9. Anastasiou E, Feinberg A, Tovar A, Gill E, Vilcassim MJR, Wyka K, Gordon T, et al. Secondhand smoke exposure in public and private high-rise multiunit housing serving low-income residents in New York City prior to federal smoking ban in public housing,

10. Hewett MJ, Sandell SD, Anderson J, Niebuhr M. Secondhand smoke in apartment buildings: renter and owner or manager perspectives. *Nicotine Tob Res*. 2007; 9:S39–47. doi: 10.1080/14622200601083442.

11. Køster B, Brink AL, Clemmensen IH. “Neighbour smoke”—exposure to secondhand smoke in multiunit dwellings in Denmark in 2010: A cross-sectional study. *Tob Control*. 2012; 22(3), 190–193. doi:10.1136/tobaccocontrol-2011-050393.

12. Kim J, Lee K, Kim K. Factors associated with secondhand smoke incursion into the homes of non-smoking residents in a multi-unit housing complex: A cross-sectional study in Seoul, Korea. *BMC Public Health*. 2017;17(1):739. doi: 10.1186/s12889-017-4774-x.

13. Leung LT, Ho SY, Wang MP, Lo WS, Lam TH. Exposure to secondhand smoke

from neighbours and respiratory symptoms in never-smoking adolescents in Hong Kong: A cross-sectional study. *BMJ Open*. 2015;5(11):e008607. doi: 10.1136/bmjopen-2015-008607.

14. Smoking and Tobacco Products Usage (Control) Act, 2005 [Internet], 2005 Mar 15 (cited 2022 Jun 11) (Bangladesh). Available from: <https://www.banglajol.info/index.php/SSR/article/view/56516/39441>

15. World Health organization. Making a difference: Tobacco control in Bangladesh [Internet]. 2017 (cited 2022 Apr 03). Available from: <https://www.who.int/bangladesh/news/detail/15-01-2017-making-a-difference-tobacco-control-in-bangladesh>

16. World Health Organization. WHO framework convention on tobacco control (FCTC) [Internet]. 2003 (cited 2022 Apr 03). Available from: <http://apps.who.int/iris/bitstream/handle/10665/42811/9241591013.pdf?sequence=1>

17. National Housing Authority. National housing policy 2016. Available from: <http://nha.portal.gov.bd/sites/default/files/files/nha.portal.gov.bd/law/76f125dc_8e5e_4095_b03d_7d9ac29f842d/National%20Housing%20Policy%202016_English%20Version.pdf>

18. Centers for Disease Control and Prevention (CDC). Tobacco [Internet]. 2017 (cited 2022 Apr 04). Availbale from: <https://www.cdc.gov/biomonitoring/tobacco.html>

19. Flouris AD, Metsios GS, Carrillo AE, Jamurtas AZ, Gourgoulianis K, Kiropoulos T, et al. Acute and short-term effects of secondhand smoke on lung function and cytokine production. *Am J Respir Crit Care Med*. 2009;179: 1029–1033.

20. Metsios GS, Flouris AD, Angioi M, Koutedakis Y. Passive smoking and the development of cardiovascular disease in Children: A systematic review. *Cardiol Res Pract*. 2011; 2011: 587650.

21. National Center for Chronic Disease Prevention and Health Promotion (US) Office on Smoking and Health. *The health consequences of smoking—50 years of progress: A report of the Surgeon General*. Atlanta (GA): Centers for Disease Control and Prevention (US); 2014.

22. He J, Vupputuri S, Allen K, Prerost MR. Passive smoking and the risk of coronary heart disease-a meta-analysis of epidemiological studies. *N Engl J Med*. 1999;340:920–926.

23. Oono IP, Mackay DF, Pell JP. Meta-analysis of the association between secondhand smoke exposure and stroke. *Journal of Public Health*. 2011;33(4):496-502. doi:10.1093/pubmed/fdr025.

24. U.S. Department of Health and Human Services. Let’s make the next generation tobacco-free: Your guide to the 50th anniversary Surgeon General’s report on smoking and health. Atlanta: U.S. Department of Health and Human Services, Centers for Disease Control and Prevention, National Center for Chronic Disease Prevention and Health Promotion, Office on Smoking and Health, 2014.

25. Jinot J, Bayard S. Respiratory health effects of exposure to environmental tobacco smoke. *Rev Environ Health*. 1996;11:89–100. doi: 10.1515/reveh.1996.11.3.89

26. World Health Organization. Women's exposure to second-hand smoke: A serious health concern [Internet]. 2012 (cited 2022 Jun 05). Available from: <https://www.who.int/china/news/detail/06-11-2012-women-s-exposure-to-second-hand-smoke-a-serious-health-concern>

27. Jallow IK, Britton J, Langley T. Prevalence and factors associated with exposure to secondhand smoke (SHS) among young people: a cross-sectional study from the Gambia. BMJ Open. 2018;8(3):e019524. Published 2018 Mar 14. doi:10.1136/bmjopen-2017-019524.

28. World Health Organization, Regional Office for South-East Asia. Global Youth Tobacco Survey (GYTS): Bangladesh report, 2013. New Delhi: WHO-SEARO, 2015.
