# Supplementary material for: Secondhand smoke exposure and associated factors among city residents living in multiunit housing in Bangladesh
Source: PLoS One. 2023 Sep 21;18(9):e0291746. doi: 10.1371/journal.pone.0291746 (PMC10513191; doi:10.1371/journal.pone.0291746)
Supplement: S2 File — (DOCX) [file pone.0291746.s002.docx]

**Questionnaire of the study**

| **Basic Information** | | |
| --- | --- | --- |
| **Ques no.** | **Questions** | **Options and code** |
| 1 | Respondent’s sex | Male…………………………….....1  Female…………………..……..….2 |
| 2 | How old are you? | 18 to 39 years….……………....…..1  40 to 59 years…………..…........…2  60 years and above……….…....….3 |
| 3 | What is your highest level of education? | Primary…………..….………..…...1  Secondary……….…….…..…........2  Higher secondary…….……..….....3  Tertiary…….…..……….........…....4 |
| 4 | What is your marital status? | Single………………….....………..1  Married………………….…..….....2  Divorced/widowed………..…..…..3 |
| 5 | What is your occupation? | Service holder…………..........…....1  Business…….………..…………....2  Student………………....…….........3  Retired…………….………...….....4  Housewife……………………........5  Others………………………….…..6  Please specify_____________ |
| 6 | What is your religion? | Muslim……………..…..…..…......1  Hindu…………………….…......…2  Christian……………….………….3  Buddhist………….………………..4 |
| 7 | What is your monthly family income? | Below 50,000 BDT….…….…..…..1  50,000 to 99,000 BDT…………….2  100,000 BDT and above…...…..….3 |
| 8 | What type of housing complex do you live in? | Government……………….…….…1  Private………………………..……2 |
| 9 | Which divisional city do you live in? | Dhaka city………………..…..….…1  Chattogram city………….…………2  Rajshahi city….……………...….….3  Khulna city………………...………4  Sylhet city…………….……………5  Barishal city…………………....…..6  Rangpur…………………..………7 |
| **Smoking Behaviours** | | |
| 10 | On how many days during the past 30 days did you smoke cigarettes/bidis? | _______days |
| 11 | Does any member of your family smoke cigarettes/bids? | Yes…………………………..…..1  No………………………...….….2 |
| **Secondhand Smoke Exposure** | | |
| 12 | On average, how long did you stay at home per day during the past 30 days? | _____________hours/day |
| 13 | On how many days during the past 30 days, did you get the smell of cigarettes/bidis from the following places? | (a) Own flat________________days  (b) Next flat________________days  (c) *Common spaces_________days  (d) Next building____________days |
| 14 | How often did you get the smell of cigarettes/bidis per day on average during the past 30 days? | (a) Own flat_____________hours/day  (b) Next flat_____________hours/day  (c) *Common spaces______hours/day  (d) Next building_________hours/day |
| **Note: ***Common spaces include waiting space, parking area, basement, stairs, roof, doorway and main entrance of the housing complex. Interviewers are instructed to read out these places when the issue of common spaces will come. | | |
