# Supplementary material for: Secondhand smoke exposure and associated factors among city residents living in multiunit housing in Bangladesh
Source: PLoS One. 2023 Sep 21;18(9):e0291746. doi: 10.1371/journal.pone.0291746 (PMC10513191; doi:10.1371/journal.pone.0291746)
Supplement: S4 File — (DOCX) [file pone.0291746.s004.docx]

# Codebook

| **Variables** | **Values** |
| --- | --- |
| **Basic Information** |  |
| Sex | 1=Male  2=Female |
| Age | 1=18-39 years  2=40-59 years  3=60+ years |
| Education | 1=Primary  2=Secondary  3=Higher secondary  4=Tertiary |
| Marital status | 1=Single  2=Married  3=Divorced/widowed |
| Occupation | 1=Service holder  2=Business  3=Student  4=Retired  5=Housewife  6=Others |
| Religion | 1=Muslim  2=Hindu  3=Christian  4=Buddhist |
| Monthly family income | 1=<50,000 BDT  2=50,000-99,000 BDT  3=≤100,000 BDT |
| Housing type | 1=Government  2=Private |
| Divisional city | 1=Dhaka city  2=Chattogram city  3=Rajshahi city  4=Khulna city  5=Sylhet city  6=Barishal city  7=Rangpur city |
| Respondent’s smoking status | 1=Smoker  2=Non-smoker |
| Family smoking status | 1=Yes  2=No |
| Length of stay at home | 1=≤12 hours  2=>12 hours |
| SHS in own flat (Recode) | 1=Yes  0=No |
| SHS from next flat (Recode) | 1=Yes  0=No |
| SHS from common spaces (Recode) | 1=Yes  0=No |
| SHS from next flat (Recode) | 1=Yes  0=No |
| Frequency of SHS in own flat | 1=1 to 3 times  2=4 to 7 times |
| Frequency of SHS from next flat | 1=1 to 3 times  2=4 to 7 times |
| Frequency of SHS from common spaces | 1=1 to 3 times  2=4 to 7 times |
| Frequency of SHS from next building | 1=1 to 3 times  2=4 to 7 times |
| Overall SHS within the MUH complex | 1=Yes  0=No |
